# 

**Published:** 1827-02

**Authors:** 


### MONTHLY LIST OF MEDICAL BOOKS.

[*No books can be entered on this List except those sent to us for the purpose; as, in the list hitherto transmitted, the names of works have frequently been given as published, which have not appeared for weeks, or even months, after.*]

No. I. (to be continued Monthly,) of Medical Botany, or Illustrations and Descriptions of the Medicinal Plants of the London, Edinburgh, and Dublin Pharmacopœias, with those lately introduced into Medical Practice; including also a Popular and Scientific Description of Poisonous Plants. By John Stephenson, M.D. Graduate of the University of Edinburgh; and J. M. Churchill, Esq. Surgeon, Fellow of the Medico-Botanical Society of London.—8vo. London, 1827.

Rheumatism, and some Diseases of the Heart and other Internal Organs: considered in the Gulstonian Lectures, read at the Royal College of Physicians, May 1826. By Francis Hawkins, M.D. Fellow of St. John's College, Oxford, and of the Royal College of Physicians; and one of the Physicians to the Middlesex Hospital.—8vo. London, 1826.

A Syllabus of Surgical Lectures on the Nature and Treatment of Fractures, Diseases of the Joints, and Deformities of the Limbs and Spine: containing Descriptions of the Modes of applying twelve new Apparatuses, illustrated by twelve Plates. With Cases, showing the Advantages arising from the Plans of Treatment recommended by J. Amesbury, F.S.A. F.L.M.S. Surgeon to the South London Dispensary, &c.—8vo. London, 1827.

A Treatise on the Diseases of Children; with Directions for the Management of Infants from the Birth. By the late Michael Underwood, M.D. Eighth Edition, revised, with Notes and Observations, by Samuel Merriman, M.D. F.L.S. Corresponding Member of the Imperial and Royal Academy of Sciences at Siena.—8vo. London, 1827.

An Essay on Medical Education. By Wm. Barrett Marshall, an Assistant Surgeon in the Royal Navy.—12mo. London, 1827.

A Physiological Enquiry respecting the Action of Moxa, and its Utility in Inveterate Cases of Sciatica, Lumbago, Paraplegia, Epilepsy, and some other Painful, Paralytic, and Spasmodic Diseases of the Nerves and Muscles. By William Wallace, m.r.i.a. &c. &c. Surgeon to the Charitable Infirmary of Dublin, and to the Infirmary for the Treatment of Rheumatism and Cutaneous Diseases in that City; Lecturer on Semeiology and Clinical Surgery.—8vo. Dublin, 1827.

An Essay on the Use of Chlorurets of Oxide of Sodium, and of Lime, as powerful Disinfecting Agents; and of the Chloruret of Oxide of Sodium, more especially as a Remedy of considerable efficacy in the Treatment of Hospital Gangrene; Phagedenic, Syphilitic, and ill-conditioned Ulcers; Mortification; and various other Diseases. (Dedicated by permission to the Right Honourable Robert Peel.) By Thomas Alcock, Member of the Royal College of Surgeons in London; Member of the Medical and Chirurgical Society, &c. &c.—8vo. London, 1827.

The Medical Student. No. I.—8vo. London, 1827.

### METEOROLOGICAL JOURNAL,

### NOTICES.

*Papers have been received from* Mr. Chandler, Dr. Gregory, Mr. Kingsley, Mr. Hunter, Mr. Churchill, Mr. Thomson, Mr. Green, *and* Mr. Shaw.

*The Communication signed* "An Apothecary" *is not fitted for insertion in this Journal.*

*The case of* "*Diarrhœa*" *is not of sufficient interest for publication.*

*We shall take an opportunity of complying with* Mr. Alison's *wishes.*

